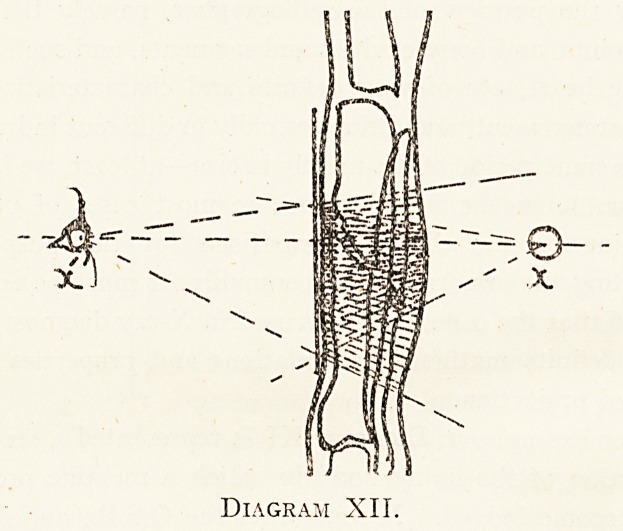# The Radiographic Centroscope and the Radiographic Episcope

**Published:** 1914-09

**Authors:** William Cotton


### THE RADIOGRAPHIC CENTROSCOPE AND THE RADIOGRAPHIC EPISCOPE.

BY

William Cotton, M.D.

---

In the diagnostic use of X-rays three degrees of information are to be obtained.

In the first degree there is determined from attentive observation of the X-ray picture (in comparison with the normal appearance) the presence or absence of what we are led to expect from the history and symptoms of the case. Such are the presence or absence of concretions or calculi or foreign bodies, the multiplication or transposition of the parts depicted, marked alterations of outline, alterations of the texture of the parts from gouty or rheumatoid or tubercular or other deposit and infiltration, the presence of fracture in the form of fissure or of fragments, and the like. This is the method of simple inspection.

In the second degree there is determined, from an exact knowledge of the position of the radiographic centre of emission to the object and to the picture plane at the time of exposure, the direction in which the parts depicted lie within the body. This is the method of perspective.

In the third degree there is determined from two or more X-ray pictures (taken according to certain rules from different points) the relative or actual position within the objects of the parts depicted. This may be called the method of stereometry, and includes all the methods of X-ray stereoscopy and localisation.

Of these three methods our greatest clinical use has been made of the first and third. The second—the method of perspective—has been almost entirely neglected and misunderstood, from an initial and almost universally prevailing error of viewing the X-ray picture from the wrong side; this mistake, being originally made in Germany, needs a lot of shifting.

Except in such a case as the case of a projectile which has split asunder into fragments within the body (*e.g.* from impact on a bone), the clinical evidence from simple inspection is merely confirmatory or negative. But when the diagnosis of direction is made, on correct principles of perspective, we get fresh radiographic evidence of positive value, not obtainable from any other method of physical examination, which evidence may obviate any necessity of further appeal to a second exposure in accordance with the methods of X-ray stereoscopy and localisation.

Perspective may be defined here as that branch of geometry which treats of the appearance of objects to an observer's eye, as distinguished from their actual shape. It actually deals with the principles of picture making and picture viewing. According to the rules of ordinary perspective, a picture is any representation on a surface usually flat, of any near object in space, by straight lines which radiate, or may be supposed to radiate, from a fixed point called the centre of projection. This description comprehends visible shadows thrown by a small lighted candle on a screen within an ordinary room, ordinary photographs, and pinhole photographs of near objects, ordinary drawings, and X-ray pictures; the centre of projection being respectively the lighted candle, the optical centre of the photographic lens, the pinhole of the pinhole camera, the optical centre of the refracting media of the observer's eye, and the anticathodal centre of emission of the X-ray focus tube in action, as the case may be. Further, according to these rules, the original position of the centre of projection in reference to the picture and in reference to the object (at the time of making the picture), is the only correct station point of the observer's one or other eye, when he seeks to observe the corresponding parts in the picture and in the object (supposed transparent) under the same angles point over point, " in perspective " as it is called. This last is in fact the fundamental theorem of ordinary perspective, and this original position of the centre of projection I will call, once for all, the original station point O.S.P. ; and my very paradoxical thesis I hope to exemplify on the present occasion by starting from the simplest case, namely that of the ordinary shadow.

By wrong side of an X-ray picture, therefore, is meant that side of the X-ray picture remote from the focus tube at the time of making the picture ; that is, the image on the fluorescent screen (black on white) viewed in the ordinary way, or the X-ray photographic plate (white or clear glass on black) viewed from its glass side, and the X-ray contact print therefrom commonly so-called viewed from its printed side (black on white). Practically the X-ray fluorescent screen cannot be viewed from its right side adjacent to the O.S.P. ; but in the case of the developed X-ray photographic plate, the right side of the plate is of course the film side, and the right side of the X-ray print is of course the paper side, which is usually sufficiently transparent to be viewed therefrom, or ought to be. The prevailing and initial error in the interpretation of any single X-ray picture is the viewing of them from the wrong side, as above defined, let us say from a station point (which may be called once for all the corresponding station point—C.S.P.) as far on the wrong side of the picture as the O.S.P. was on the right side at the time of exposure, such that the C.S.P. on the wrong side is directly opposite to the O.S.P. on the right side. As I hope to show, this fundamental error involves another, namely of getting

DIAGRAM I.

DIAGRAM II.

DIAGRAM III.

DIAGRAM IV.

on to the wrong side of the object, that side of the object namely that if visible and transparent would show the parts under lesser angles than the parts would be seen under in the X-ray picture.

Diagram I shows a shadow by ordinary light of a spear-shaped object thrown on a screen such as a window-blind. The object is some distance from the screen, and its long axis lies oblique thereto. The observer on the outside of the window has his eye at the C.S.P., the lighted candle being of course at the O.S.P. If he fires at any point of the shadow he is bound to miss that point in the object unless he fires directly at the candle or unless the part of the shadow he aims at has the corresponding part of the object in contact with it.

Further, if the window blind is pulled up he will see every part in the object under smaller angles with the line joining the candle and his eye than he saw each part under in the shadow on the blind when it was down.

But if he could put his eye where the lighted candle is without extinguishing it or hurting himself, or if his eye was itself luminous, then with his eye at the O.S.P. of the candle, to him when the blind was down the object would exactly cover no more and no less point for point its shadow on the blind—that is, the object and its image would be "in perspective." As we shall see presently, the shadow on the blind viewed from the C.S.P. is in form and tone exactly the case of the X-ray fluorescent screen, and of the X-ray print.

Diagram II shows a further development of the same idea. A second lighted candle is now placed at the C.S.P., towards the right-hand side of the diagram, and the surface of the screen is supposed to be highly glazed like the glazed surface of a P.O.P. X-ray print, and sufficiently so to act as a plane mirror, as may easily be done. When the observer sees the reflection of this second lighted candle fall over that point of the screen which he wishes to hit in the object, then he may be sure (from the laws of reflection of light in the case of a plane mirror) that his eye and the pistol and the point of the object he wishes to hit and its image on the window blind are all in line. This, in fact. is the principle and one of the uses of an instrument called the centroscope, to which further allusion will be made.

With objects of this simple kind X-rays would be unnecessary as long as there was a candle about. In Diagram III I have drawn the usual case of an object to be X-rayed, namely a visibly opaque external object transparent to X-rays, containing concealed internally an object such as that shown opaque to X-rays.

I have drawn this compound object from its back and its front—on the right hand the box is intact ; on the left hand I have displayed the internal spear-shaped object to be X-rayed. This latter object is situated in one of the diagonals of the box as shown. The surface markings on the back and front and sides of the box are intentionally reminiscent of some of the superficial markings of the human trunk.

Diagram IV shows the same idea as in Diagram II, worked out more in detail, in the case of an X-ray tube irradiating a compound object (as in the last diagram) upon the fluorescent screen ; some of the superficial and internal structures of the object are partially indicated, and the internal object is clearly shown. This is also the case of the X-ray contact print. The compound object is being taken ventrodorsally.

Supposing it to be the case of the fluorescent screen, and the observer to have his eye at the C.S.P., I have in this present diagram indicated a second form of the principle of the centroscope. A small circular plane mirror with a central aperture (a plane retinoscope stripped so as to have

Diagram V.

Diagram VI.

Diagram VII.

its silvered side flush with the plane of the fluorescent screen would do) is placed round the part whose true direction through the O.S.P. is to be ascertained. When the image of a small luminous flame is observed to disappear over the central hole of the retinoscope, then we know (from the laws of reflection of light from a plane mirror) that the candle, the hole in the retinoscope (or centroscope), and the point selected in the object itself are all in line with the O.S.P.

It is not now a question of putting a bullet in, but rather of getting one out ! The practical use of such an instrument as the centroscope is probably limited to the case of a foreign body, when only one X-ray picture is obtainable. Further particulars will be found elsewhere, with illustrated diagram. [1]

Diagram V is the same as Diagram I, but drawn from a diametrically opposite side of the scene of operations. It shows the shadow of the same object in the same relation to the screen and to the source of illumination. So we see the shadow on the right side of the screen, that is on the inside of the window blind. This diagram is necessary as an introduction to the next.

Diagram VI shows the observer with his eye at the O.S.P. The object with its face towards him has been X-rayed as before ventrodorsally, but the picture is a developed X-ray photographic plate (white on black) with the film side turned towards the observer, just as according to universal practice the film side was turned to the X-ray centre of emission at the time of exposure. Unfortunately, the visible object is non-transparent, and the observer cannot see the picture of the box on account of its being between him and his picture.

Now there are several things he could do. He could put the object to one side, and look alternately at it and at his white on black primary picture on glass, preserving the proper position at the O.S.P. of his eye to each (the object and the picture) alternately. Or he might make a carbon print by contact (a green or blue or yellow or red, etc., on white high lights) from his glass plate (white on black), taking care to make only one reverse, and view his object and carbon print alternately in the same way. Or he might reduce his X-ray photograph in size by ordinary photographic means, and have it mounted as a transparency, and hold it at its corresponding distance between his eye at the O.S.P. and the ventral surface of its object if taken ventrodorsally (or its dorsum if taken dorsoventrally).

I have, however, experimentally devised a better way (see Diagram VII). If (with his one eye at the O.S.P., in regard to the object) there be interposed between the object and the eye a transparent flat thin sheet of polished glass inclined at an angle of 45° to the principal visual axis as shown, then with his X-ray print (black on white) to one side at a distance from the centre of the inclined transparent reflector equal to what the glass photographic plate had at the time of exposure, a reversed virtual image (black on white) will be formed on the original site of the glass photographic plate. Mentally this reflected X-ray image is referred to the visible surface of the object adjacent to the observer's eye, and under the proper perspective angles, so that the outlines can be readily pencilled on the surface, as indicated in the diagram. What he sees, his combined view, is indicated on the screen to the left of the diagram—he sees both the X-ray image and the visible part of his object in proper perspective register; while his one eye refers the X-ray image to the near surface of the object, he sees every part under the correct perspective angles, correct alike in form and in tone.

There has recently been much controversy in regard to the position of the stomach according to X-ray pictures, and

Diagram VIII.

Diagram IX.

according to anatomical investigation. Now, no internal organ can have its position determined from any single X-ray picture; we can only tell therefrom the direction its boundaries will be found in when seen "in perspective." Diagram VII has been published before, but I cannot refrain from introducing it here, as a pictorial record of an actual experiment; it shows the only correct method, by means of an episcope, of outlining on the surface of the body such an outline as the organ would present to the observer's eye at the O.S.P., if the organ were actually visible.

Diagram VIII shows the same experiment in horizontal section or plan. Figure I shows the picture making, and Figure II shows the picture viewing. The transparent inclined reflector (which is very appropriately called the radiographic episcope) is shown, enclosed in a kind of box or camera obscura, with two eye-pieces, the one for centring the visible object and viewing the combination picture, the other eye-piece is for centring the X-ray print (black on white). Either eye-piece can be used with either eye.

Diagram IX shows this form of "enclosed" episcope (in isometric perspective with scale). There is a slit with a hinged flap on the top of the box for inserting or removing the transparent reflector, which should be of thin microscopical cover glass to avoid a double reflection from its two surfaces, but not too thin to be broken in manipulating or cleaning; it costs a few shillings.

In speaking of the second degree of information to be obtained from the diagnostic use of X-rays, I have said we must have first "an exact knowledge of the position of the radiographic centre of emission to the object and to the picture plane at the time of exposure." I have elsewhere shown how very simple this knowledge can be secured.[2]

Diagram X shows a "simplified" and quite efficient episcope, a circular cover glass of about $\frac{7}{10}$ of an inch in radius, mounted in the cleft end of a wooden match; it costs a few pence. The size is calculated on certain assumptions as to the dimensions of the eye-ball, and as to the position of the optical centre of the refracting media, etc., and gives a "clearance" of an inch. No doubt smaller ones would be practically accurate. The inner edge of the disc may be conveniently inclined against the inner canthus of the eye employed.

DIAGRAM X.

In using any episcope it must be remembered that the direct image of the uncovered human body is as a rule much brighter and stronger than the partially reflected image of the X-ray print. Just as the pianoforte accompaniment must be played "piano" so as not to drown a feeble soloist, so the object directly viewed must be well in the shade. As suggested long ago[3] in the case of an instrument used in drawing microscopic objects called a "parallel plate reflector" (but really a "simplified" cover glass episcope), the glare may be lessened by tinting the glass. I have little doubt these optical instruments will all be traced ultimately as far-flown chips from Helmholtz's workshop!

I believe I was the first to suggest and work out correctly the application of ordinary perspective to X-ray pictures. All medical authority is against my point of view. Unfortunately Röntgen, unlike Helmholtz, was not himself a medical man, nor did he understand medical or surgical needs and limitations. All were misled, and have continued to be misled, by taking the X-ray negative to correspond with the photographic negative, and the

X-ray print with the photographic print in form, as well as in tone.

The most serious objection I have met with is that the human body and its organs is not a collection of regular geometrical figures. While this is so, it must be acknowledged firstly, that the parts of the body likely to come under the purview of the radiographer, namely the bones and joints, and bony cavities and segments, and such organs as the heart, are of very definite and characteristic shape and arrangement, and differ normally in different individuals of the same period of life merely in size—at least, we have to assume so in the first instance in most cases of physical examination for clinical comparison for the purpose of detecting the abnormal ; and secondly, it must be acknowledged that the cone of X-rays used in X-ray diagnostics has very definite mathematical relations and properties in the way of projection.

For example, in Diagram XI is represented a section of a portion of the living body in which a metallic projectile has become lodged. X represents the O.S.P., and X [1] the C.S.P., where the observer has his eye. The dotted lines diverging from X represent the conical space in which the bullet lies ; the dotted lines diverging from X the conical space in which the unwary observer at C.S.P. may judge

Diagram XI.

the object to be. This, of course, is a case where the centroscope may usefully be employed.

Again, in Diagram XII is represented a vertical section of a fracture of both bones in a swollen limb ; X and X[1] are as before the O.S.P. and the C.S.P. respectively. Now as indicated by the dotted lines, the observer at X[1], judging

DIAGRAM XII.

by the visible portions of the limb, must have a very exaggerated idea of the extent of the lesion, as compared with what an observer will have with his eye at X, and enjoying in some way a perspective view from O.S.P.

Putting aside such an abortive monstrosity as the instrument known as the orthodiagraph, by the use of parallel X-rays (tele-Röngtenography) instead of widely-divergent X-rays from a focus tube near the object taken, the practical clinician, especially the practical surgeon (who needs to work within arm's length), will lose more than he gains. This being so, I am inclined to think that till the day comes (it may of course be to-morrow) when we will be able to photograph by means of a suitable pin-hole camera and some suitable sensitive film, concealed objects by the secondary rays they emit when irradiated from a focus tube, the episcope and the principles of correct perspective may lead to great advances in the clinical uses of X-rays as a diagnostic, both medical and surgical.

I cordially recommend the whole subject to the attention of what I may call the younger generation of X-ray workers; clinical radiography still needs a Laennec or a Skoda. Correct theory tends to correct practice, and incorrect theory to fallacious practice; with the former the practical man may go wrong, with the latter he may go right. For example, there is the reported instance of the Viennese professor who examined a large number of patients by percussion and by X-rays, and came to the result that in no case did the outlines of the heart by these respective methods correspond. A knowledge of perspective would have enabled him to say so, without leaving his chair.

### BIBLIOGRAPHY.

Euclid, *Elements of Geometry*, Books V and VI.

J. Humphrey Spanton, *Complete Perspective Course*. London: Macmillan & Co.' 1900.

Hermann von Helmholtz, *Popular Lectures on Scientific Subjects*. Series I, Lecture VI, "The Recent Progress of the Theory of Vision." Series II, Lecture III, "On the Relation of Optics to Painting—Form." London: Longmans, Green & Co. 1901 and 1900.

### REFERENCES.

[1] "The Fluoroscopic Diagnosis of Direction by a Plane Mirror upon a Screen," *Practitioner*, 1911, lxxxvi. 725. "Design for an Elementary Radiographic Camera," *Bristol M.-Chir. J.*, 1911, xxix. 118.

[2] "The Episcope, an Optical Instrument for X-ray Use," *Arch. Röntgen Ray*, February, 1913. "Direct Combined Examination of Single Radiograph and Patient: the Episcope," *Practitioner*, 1913, xc. 1006. "The Episcope, a New Instrument for the Utilisation of the Single X-ray Print," *Arch. Röntgen Ray*, July, 1913.

[3] *Outlines of Practical Histology*. William Rutherford. London: J. & A. Churchill. 1878. Pp. 45 and 46.

## Figures and Tables

**Diagram I. f1:**
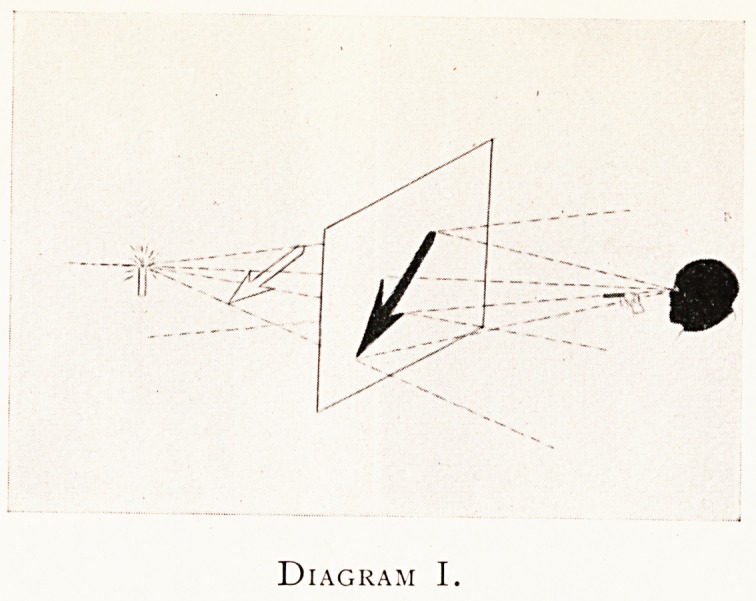


**Diagram II. f2:**
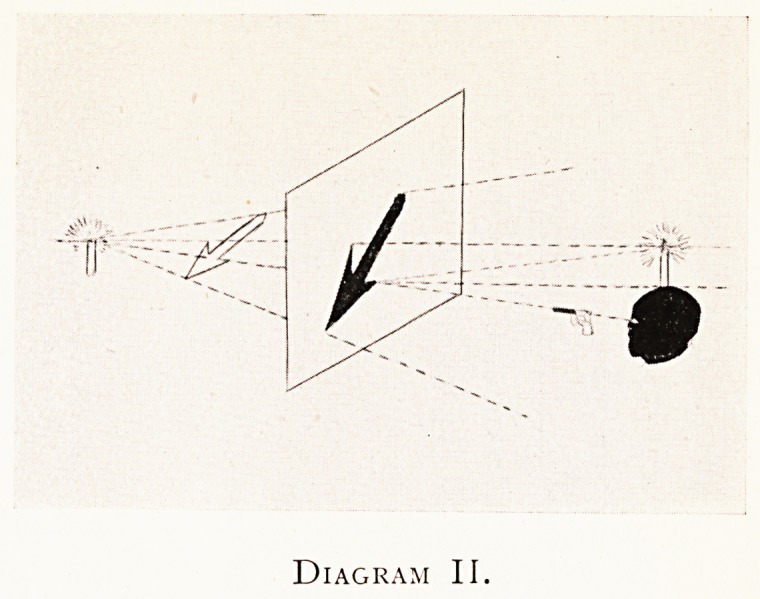


**Diagram III. f3:**
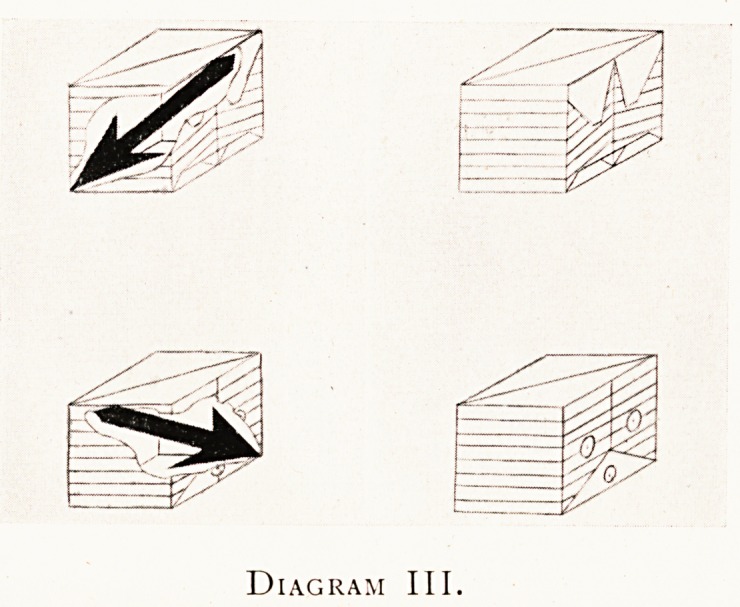


**Diagram IV. f4:**
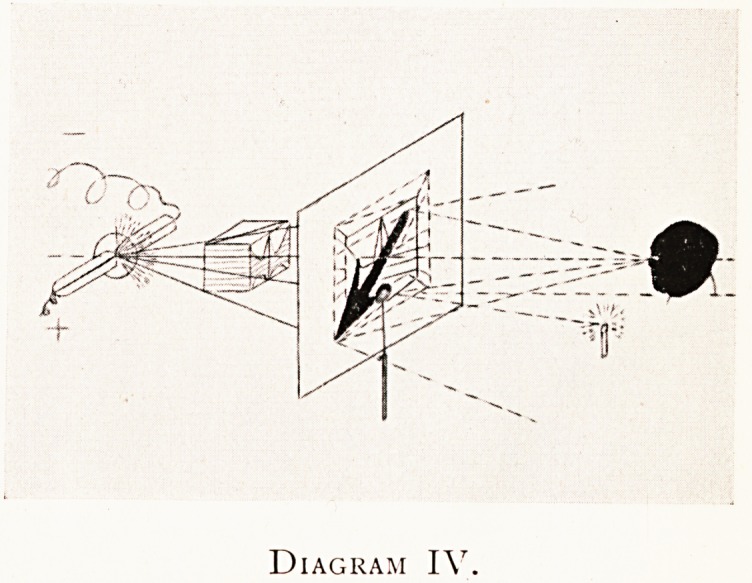


**Diagram V. f5:**
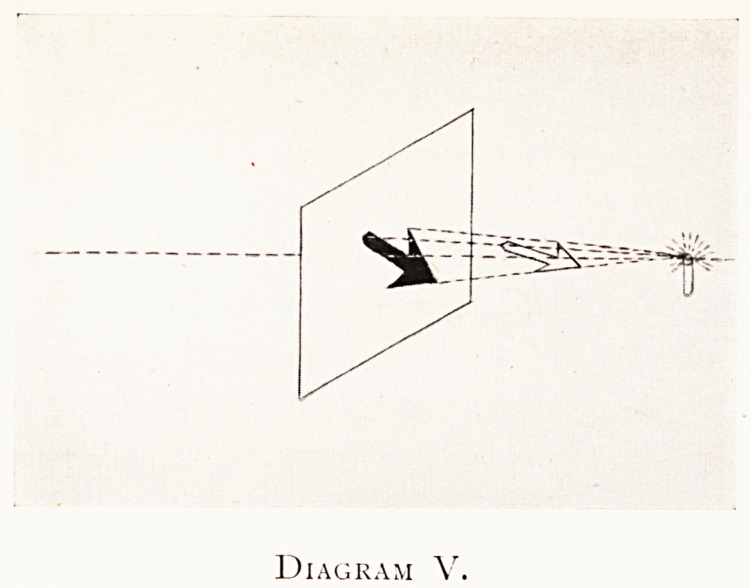


**Diagram VI. f6:**
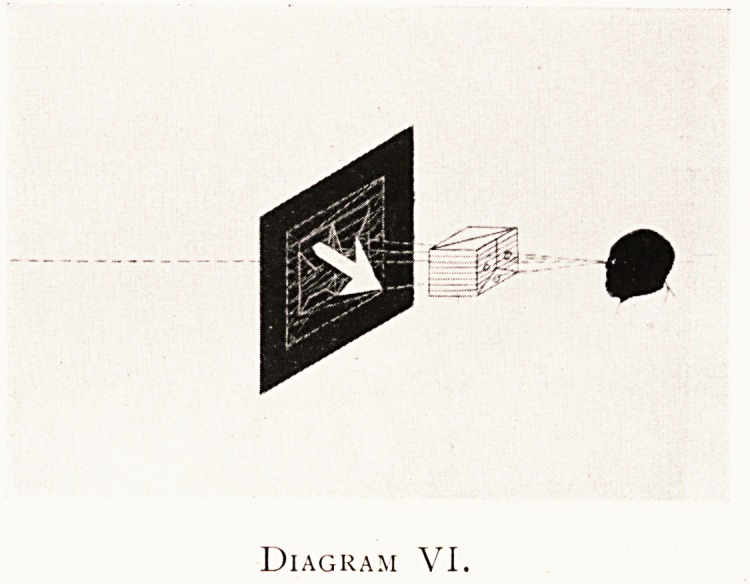


**Diagram VII. f7:**
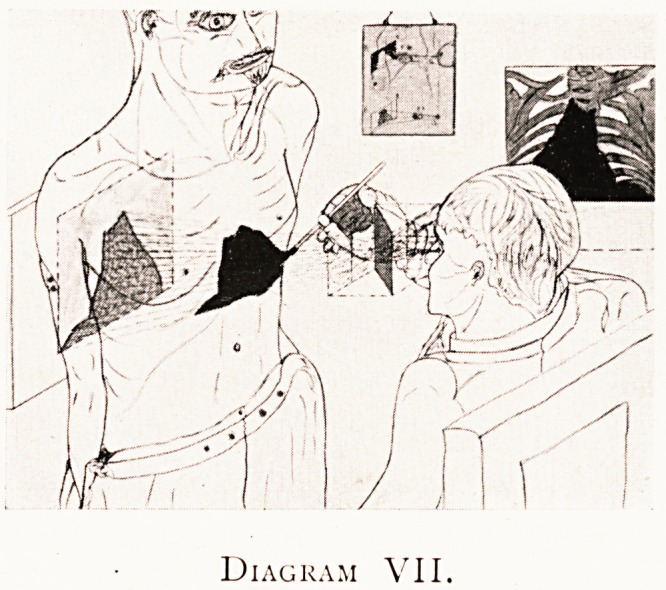


**Diagram VIII. f8:**
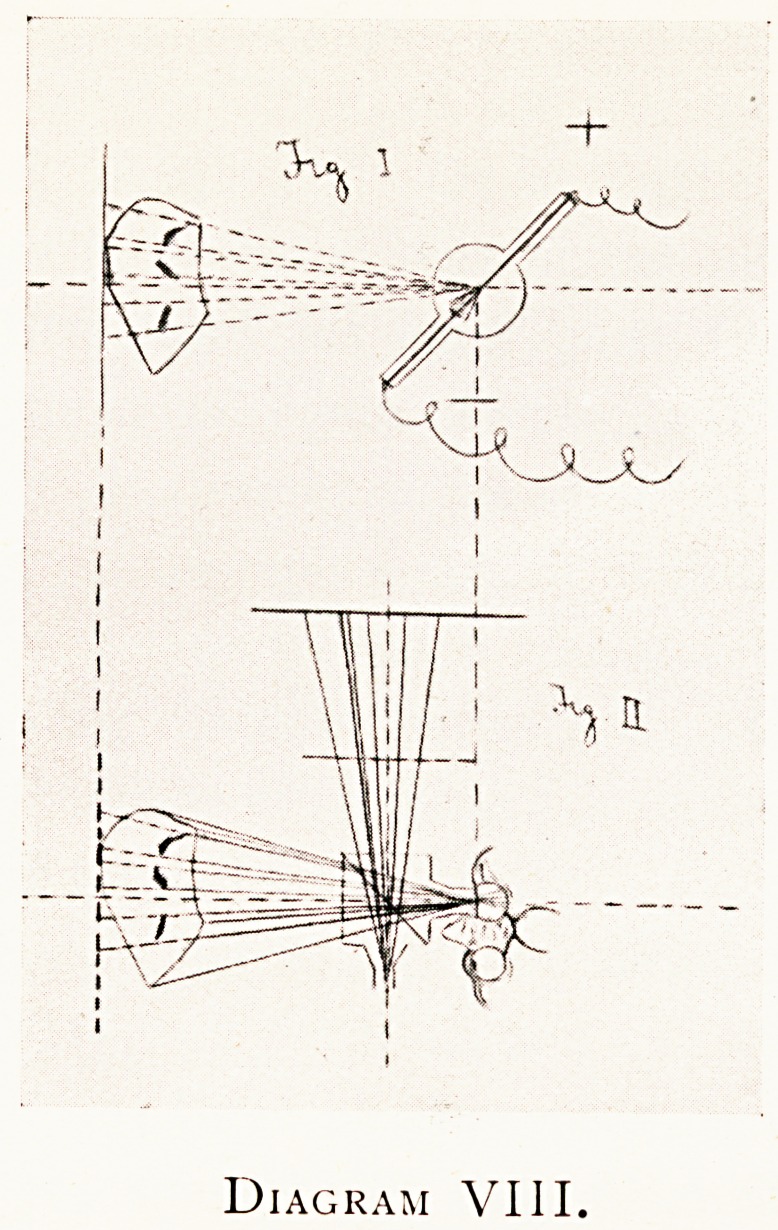


**Diagram IX. f9:**
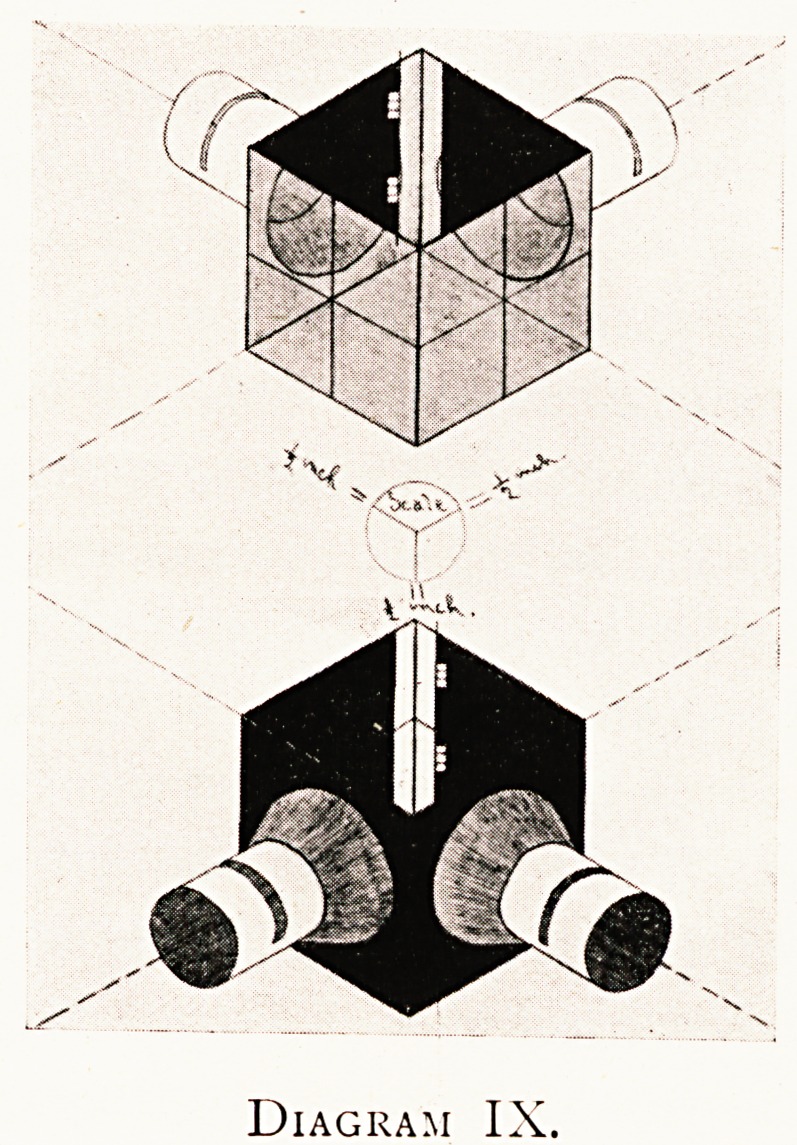


**Diagram X. f10:**
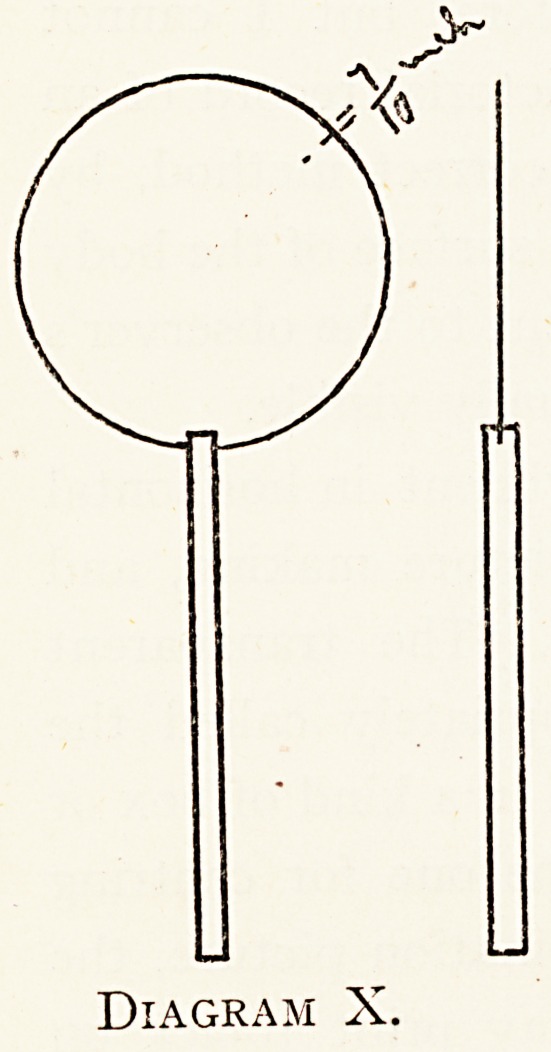


**Diagram XI. f11:**
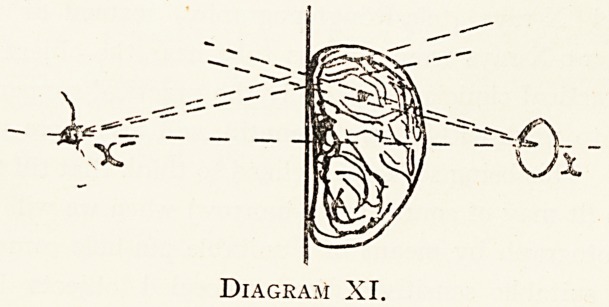


**Diagram XII. f12:**